# Surface-guided radiotherapy setup using an open-face mask for head and neck cancer; the OPEN trial approach

**DOI:** 10.1016/j.mex.2026.104186

**Published:** 2026-09-29

**Authors:** Samantha Ryan, Claire Fitzpatrick, Ciaran Malone, Margaret Tynan, Rachel Harwood, Clodagh Callagy, Ruth Woods, Sinead Brennan, Jill Nicholson

**Affiliations:** a St. Luke’s Radiation Oncology Network, St. Luke’s Hospital, Dublin, Ireland; b Applied Radiation Therapy Trinity, Discipline of Radiation Therapy & Trinity St James’s Cancer Institute, Trinity College Dublin, Ireland; c Trinity St. James’s Cancer Institute, St. James' Hospital, Dublin, Ireland

**Keywords:** Head and neck, SGRT, Open-face mask, Positioning, Immobilisation

## Abstract

Head and neck (H&N) radiotherapy requires reproducible immobilisation to maintain geometric accuracy; however, conventional thermoplastic masks are associated with patient discomfort and anxiety. Surface-guided radiotherapy (SGRT) provides non-ionising, real-time guidance for patient positioning and intrafraction monitoring from setup through to treatment completion. However, its application in H&N radiotherapy requires careful consideration of the complex and independently mobile anatomy of the face, neck and shoulders.

This MethodsX protocol accompanies the OPEN Phase III randomised control trial and describes the technical and clinical workflow for the SGRT-supported open-face immobilisation arms. It provides a reproducible framework for centres seeking to implement the OPEN approach.

• Initial alignment is performed using SGRT postural video guidance based on the patient’s external contour.
• A facial setup ROI is used for head positioning.
• A larger monitoring ROI encompassing the face, neck and upper thorax is then used to refine lower neck and shoulder position while maintaining facial alignment, and for continuous intrafraction monitoring during treatment.

The OPEN trial demonstrated that this workflow maintained setup accuracy, intrafraction stability and clinical efficiency while supporting open-face immobilisation. This companion protocol provides the practical detail required to reproduce the workflow in clinical practice.

## Graphical abstract

**Figure:**
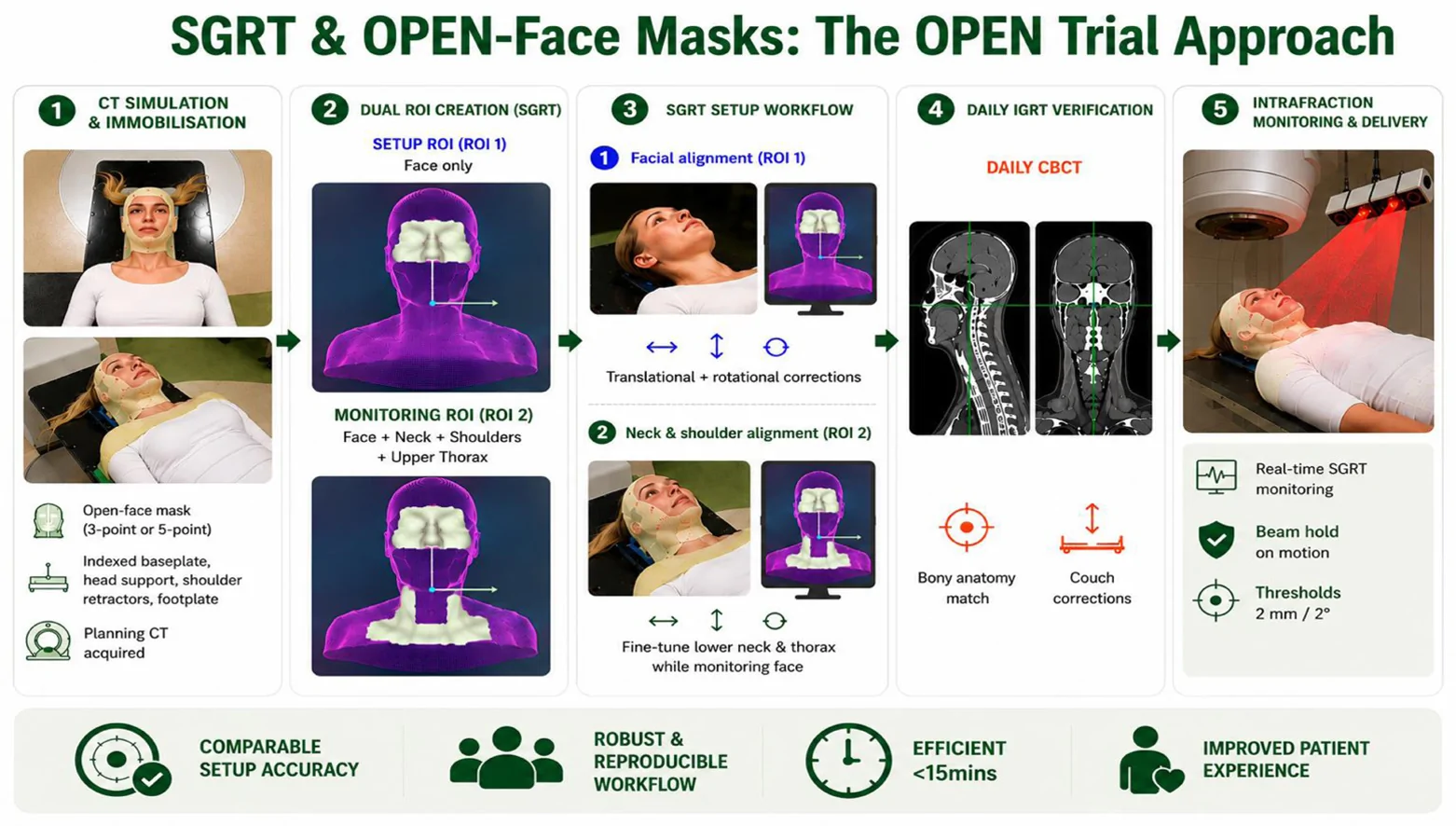

**Specifications table** (Table 1)

**Table 1:** Specifications table.

**Subject area**	*Medicine and Dentistry*
**More specific subject area**	*Radiation Oncology - Positioning and Immobilisation*
**Name of your protocol**	Nicholson J, Ryan S, Malone C et al. Trial Protocol: Open: Optimising Patient Experience in Head and Neck Radiotherapy, A phase 3 randomised control trial comparing open versus closed face masks for patients undergoing head and neck radiotherapy. [version 1; peer review: 1 approved with reservations]. HRB Open Res 2025, 8:60 (https://doi.org/10.12688/hrbopenres.14112.1)
**Reagents/tools**	TrueBeam or Trilogy treatment machines (Varian, a Siemens Healthineers company, Palo Alto, CA, USA). Orfit Industries (Wijnegem, Belgium) masks, base plate and knee support. AlignRT Advance, version 6.3 (Vision RT, London, UK) Shoulder retractors (CQ Medical, formerly CIVCO Radiotherapy, Orange City, IA, USA)
**Experimental design**	*Comparison of conventional closed-face masks with SGRT-supported open-face masks with respect to setup accuracy and patient experience.*
**Trial registration**	ClinicalTrials.gov Identifier: NCT06327139
**Ethics**	*Full prospective ethical approval was granted by the local hospital research ethics committee in September of 2023.* *All patients received a full explanation of the study and provided written informed consent prior to enrolment in the OPEN trial.*
**Value of the Protocol**	*Open-face masks improve patient comfort while maintaining setup accuracy* *Open-face masks demonstrate a statistically significant improvement in overall patient experience* *Following implementation, the open-face SGRT workflow can be delivered within standard institutional treatment appointment times.*

## Background

Head and neck (H&N) radiotherapy requires reproducible immobilisation to maintain geometric accuracy. Closed-face thermoplastic masks remain standard practice but may cause discomfort, claustrophobia and psychological distress, affecting treatment tolerability [[1], [2], [3], [4], [5]]. Open-face masks provide a less restrictive alternative, with evidence of improved patient experience without compromising setup accuracy [6].

Previous institutional work demonstrated comparable setup accuracy with open-face masks in patients experiencing mask-related anxiety [7]. However, this approach relied on image-guided radiotherapy (IGRT) for verification and did not incorporate surface-guided radiotherapy (SGRT) for setup or continuous treatment monitoring. Lundin et al. used a mixed-methods approach in which patients experienced both open and closed-face masks [8]. Although open-face masks were perceived as less confining, preference for the open-face mask was not significant; patients reported improved positioning with the closed-face mask. These findings suggest that confidence and efficiency in setup may be as important to treatment experience as mask comfort, highlighting the need for a reproducible SGRT workflow for open-face immobilisation.

SGRT provides non-ionising, real-time surface guidance, but its application in H&N radiotherapy is challenging because the treatment region includes independently mobile structures from the face to the lower neck and shoulders [9,10]. Changes in posture, weight, tumour volume and external contour may affect agreement between real-time and reference surfaces [11]. Region of interest (ROI) selection is critical: large ROIs may average regional discrepancies, while face-only ROIs provide limited information on lower neck and shoulder position [12].

Li et al. demonstrated proof-of-principle for a dual-ROI SGRT approach using separate facial and upper-chest ROIs to reproduce neck and cervical spine curvature during H&N setup [13]. This established the rationale for regional rather than single-ROI assessment. However, some requirements for broader clinical implementation remain to be addressed, including different open-face mask configurations, integration with daily IGRT, lower neck and shoulder positioning, continuous intrafraction monitoring and workflow efficiency.

Together, these findings highlight an implementation gap: reproducible guidance for integrating SGRT with open-face H&N immobilisation across mask fabrication, patient setup, image verification and intrafraction monitoring remains limited.

To address this gap, an SGRT workflow was developed for the OPEN Phase III randomised control trial. The trial recruited 230 patients across three immobilisation arms: a closed 5-point mask without SGRT (Arm A), a 3-point open-face mask with shoulder retractors and SGRT (Arm B), and a 5-point open-face mask with SGRT (Arm C) [14,15]. The workflow uses a small facial ROI for initial head positioning, followed by a larger ROI encompassing the face, lower neck and upper thorax for positional refinement and continuous treatment monitoring.

The workflow was developed through prospective feasibility testing of three ROI strategies and subsequently implemented and evaluated within the OPEN trial using daily CBCT and SGRT monitoring. The OPEN trial reports the clinical outcomes of the three immobilisation systems. This MethodsX article provides detailed development, technical configuration and step-by-step guidance for the SGRT workflow used in the two open-face treatment arms. Its purpose is to provide practical information for centres seeking to implement SGRT-supported open-face H&N radiotherapy.

## Description of protocol

### Rationale for protocol development

The SGRT workflow was developed to support safe and reproducible implementation of the two open-face immobilisation systems used in the OPEN trial. It was designed for use within a large, high-volume radiotherapy network and therefore needed to provide accurate positioning and continuous intrafraction monitoring while remaining practical for routine clinical delivery.

The workflow was developed around three core requirements:

1. **Safety and accuracy:** to support accurate patient positioning and reliable intrafraction monitoring.
2. **Robustness and reproducibility:** to perform consistently across the anatomically complex and mobile H&N region.
3. **Efficiency:** to be deliverable within standard clinical workflows, allowing for an initial staff learning curve.

The following sections describe the OPEN workflow in the sequence required for clinical replication, from immobilisation and mask fabrication through SGRT ROI preparation, daily patient setup, IGRT verification, intrafraction monitoring and troubleshooting.

### Development and selection of the dual-ROI strategy

A prospective clinical feasibility evaluation was undertaken in four patients receiving H&N radiotherapy with open-face immobilisation to develop and refine the SGRT setup workflow. Three ROI strategies were assessed:

1. **Single large ROI:** one ROI encompassing the face, neck and upper thorax for both setup and monitoring.
2. **Two separate ROIs:** a facial ROI for setup and a separate neck/upper thorax ROI for monitoring.
3. **Dual-ROI strategy:** a facial ROI for initial setup, followed by a larger combined face, neck and upper thorax ROI for positional refinement and treatment monitoring.

All strategies incorporated SGRT postural video for initial gross positioning, with daily CBCT used as the reference standard for positional verification. Strategies were evaluated iteratively based on the magnitude of CBCT-derived setup corrections, suitability for intrafraction monitoring, workflow efficiency and feedback from the treating multidisciplinary team.

The **single large ROI** provided a simple workflow but was less sensitive to regional positional differences across the long and mobile H&N treatment region.

**The two separate facial and lower neck/thoracic ROIs** improved regional assessment but required additional switching between ROIs, which reduced workflow efficiency. **The dual-ROI** strategy retained the sensitivity of a small facial ROI for head positioning while allowing simultaneous assessment of the face, lower neck and shoulders within the larger monitoring ROI. It was therefore selected as providing the most appropriate balance between positional sensitivity, intrafraction monitoring capability and clinical efficiency for the OPEN trial.

### Open-face immobilisation equipment

• **3-point open-face mask:** incorporates three fixation points around the head and is used with shoulder retractors and a footplate to reproduce shoulder position (Fig. 1).

**Fig. 1:**
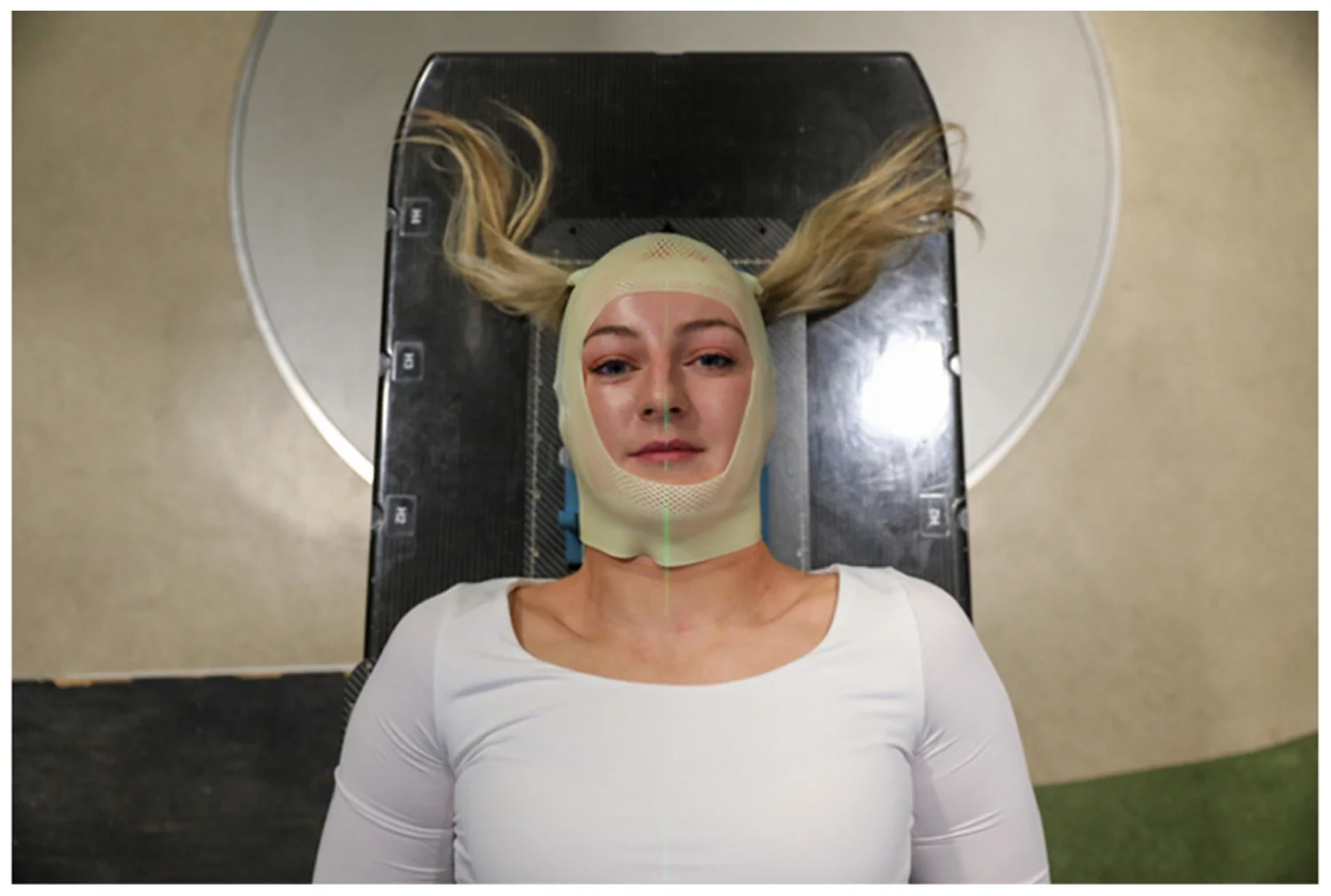
3-point open-face mask incorporating three fixation points around the head, used with shoulder retractors and a footplate to reproduce shoulder position in the absence of thermoplastic shoulder fixation.
• **5-point open-face mask:** incorporates five fixation points across the head and upper thorax, providing thermoplastic fixation of the head, neck and shoulders (Fig. 2).

**Fig. 2:**
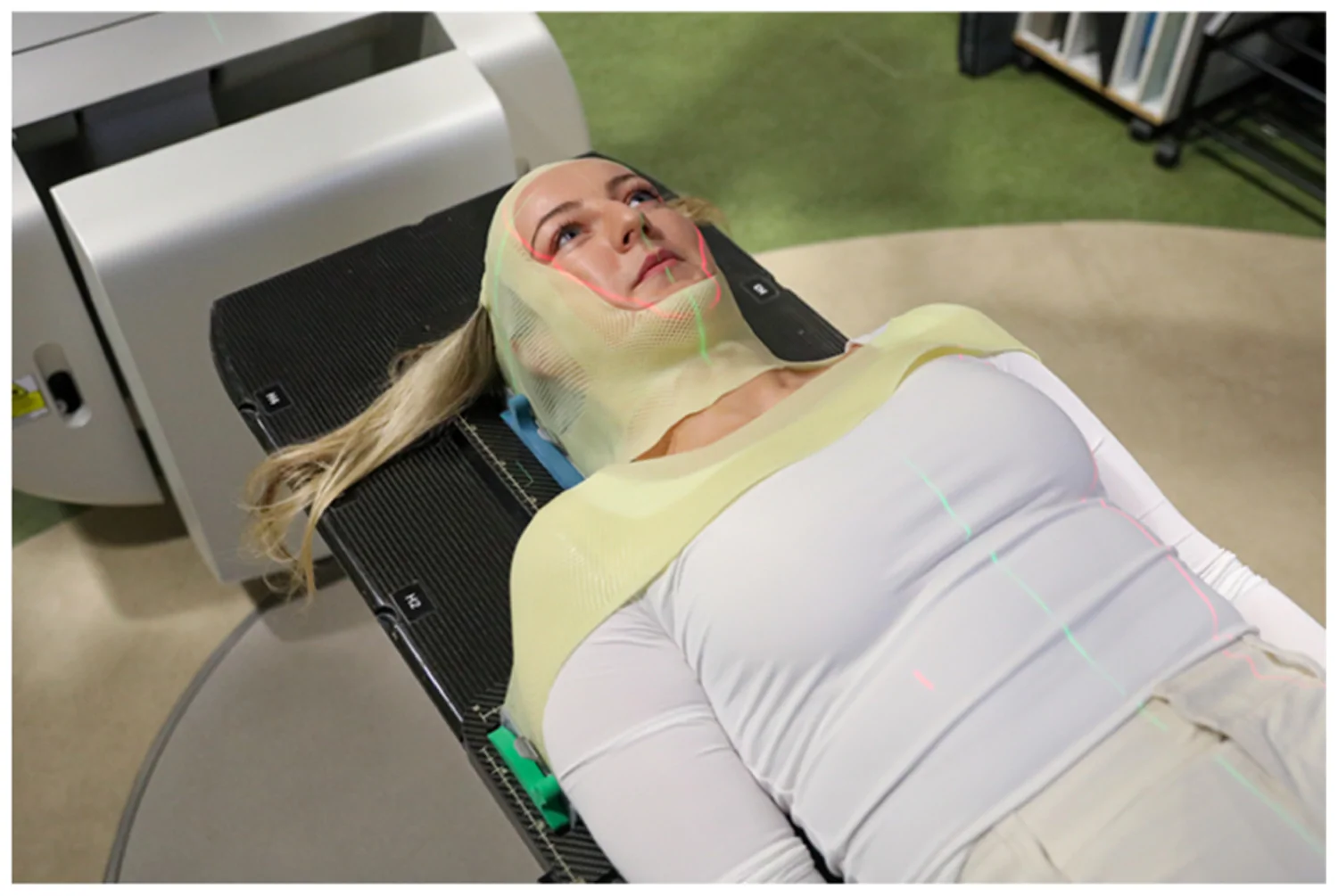
5-point open-face mask incorporating fixation across the head and upper thorax, providing thermoplastic immobilisation of the head, neck and shoulders.
• Open-face thermoplastic masks and indexed immobilisation baseplate (Orfit Industries, Wijnegem, Belgium).
• Indexed head support.
• Shoulder retractors (CQ Medical, formerly CIVCO Radiotherapy, Orange City, IA, USA) for the 3-point open-face mask.
• Knee support.
• TrueBeam or Trilogy linear accelerator with daily CBCT capability (Varian, a Siemens Healthineers company, Palo Alto, CA, USA).

### SGRT system specifications

• **SGRT system:** AlignRT Advance (Vision RT, London, UK), version 6.3.
• **Cameras:** Three Gen 5, 4 MP HD CoaXPress camera units with thermal stability were used for video-based 3D optical surface imaging.
• **SGRT quality assurance:** Daily QA was required every 24 h, with treatment inhibited if QA had expired. Monthly QA was also performed, with additional calibration following QA failure or camera movement. Initial calibration was performed by the manufacturer at installation.
• **Registration and ROI parameters:** The planning CT-derived external surface was used as the reference surface. The live patient surface was registered to the selected ROI, providing six positional offsets: vertical (VRT; anterior/posterior), longitudinal (LNG; superior/inferior), lateral (LAT; left/right), yaw/rotation (RTN), roll and pitch.
  **Setup ROIs** were used for postural alignment and **Isocentric ROIs** for positional setup and treatment monitoring. A minimum live-to-reference surface overlap of 25% was required for registration.

***1. CT simulation and immobilisation***

During CT simulation, patients were aligned using the CT sagittal laser and immobilised using either a 3-point open-face mask (Fig. 1) or a 5-point open-face mask (Fig. 2). Patients were positioned in a neutral head position where achievable. Masks were fabricated according to the manufacturer’s recommendations, with particular attention to secure fixation at the forehead and chin, and around the shoulders for the 5-point mask, while maintaining sufficient facial and thoracic exposure for SGRT.

For the **3-point open-face mask**, shoulder position was standardised using shoulder retractors secured to a footplate. Patients were positioned without shoes and held the retractor handles with their palms facing downwards, providing reproducible shoulder positioning in the absence of thermoplastic shoulder fixation.

Three radiation therapists are recommended for mask fabrication to facilitate symmetrical application and consistent facial openings, improve efficiency and minimise overstretching or puckering of the mask. The mask is heated in a water bath for the manufacturer-specified duration of 4 min at 68 °C, then dried and checked to ensure a tolerable temperature before application.

The facial opening is positioned before the mask is secured to the baseplate. One therapist maintains the position and shape of the opening around the forehead, chin and lateral aspects of the face while the remaining therapists secure the mask. The lateral fixation points are secured simultaneously to minimise asymmetric traction and unintended head roll. The superior fixation point is then secured while the forehead position is maintained. This approach also minimises excessive stretching or distortion of the facial opening.

The facial opening was standardised using anatomical landmarks to ensure consistent exposure of the facial surface for SGRT: superiorly, it extended beyond the supraorbital ridge; inferiorly, below the mouth; and laterally, beyond the outer canthus and over the zygomatic arch. Fabrication of the head and facial component was identical for the 3-point and 5-point masks.

For the **5-point open-face mask**, one therapist continued to stabilise the head and facial position while the remaining therapists moulded the inferior portion over the upper thorax and around the shoulders before securing the lower fixation points. Sufficient lower neck and upper thoracic surface remained exposed for SGRT monitoring, including the suprasternal notch and clavicles.

Simulation was then completed according to the institutional H&N radiotherapy protocol, and the planning CT dataset was used to generate the reference surface for SGRT.

***2. SGRT ROI Preparation***:

A dual-ROI configuration was prepared for treatment:

1. The Setup ROI used for initial SGRT-guided head positioning

2. The Monitoring ROI to refine neck and shoulder position following initial facial alignment and provide intrafraction monitoring during treatment delivery.

1. **Setup ROI** (Fig. 3): A small facial ROI was defined over the exposed facial surface with high topographic variation. Anatomical boundaries were defined superiorly by the supraorbital ridge, inferiorly above the mouth to minimise the influence of this mobile region, and laterally as far as permitted by the mask aperture, extending beyond the outer canthus and over the zygomatic arch.

**Fig. 3:**
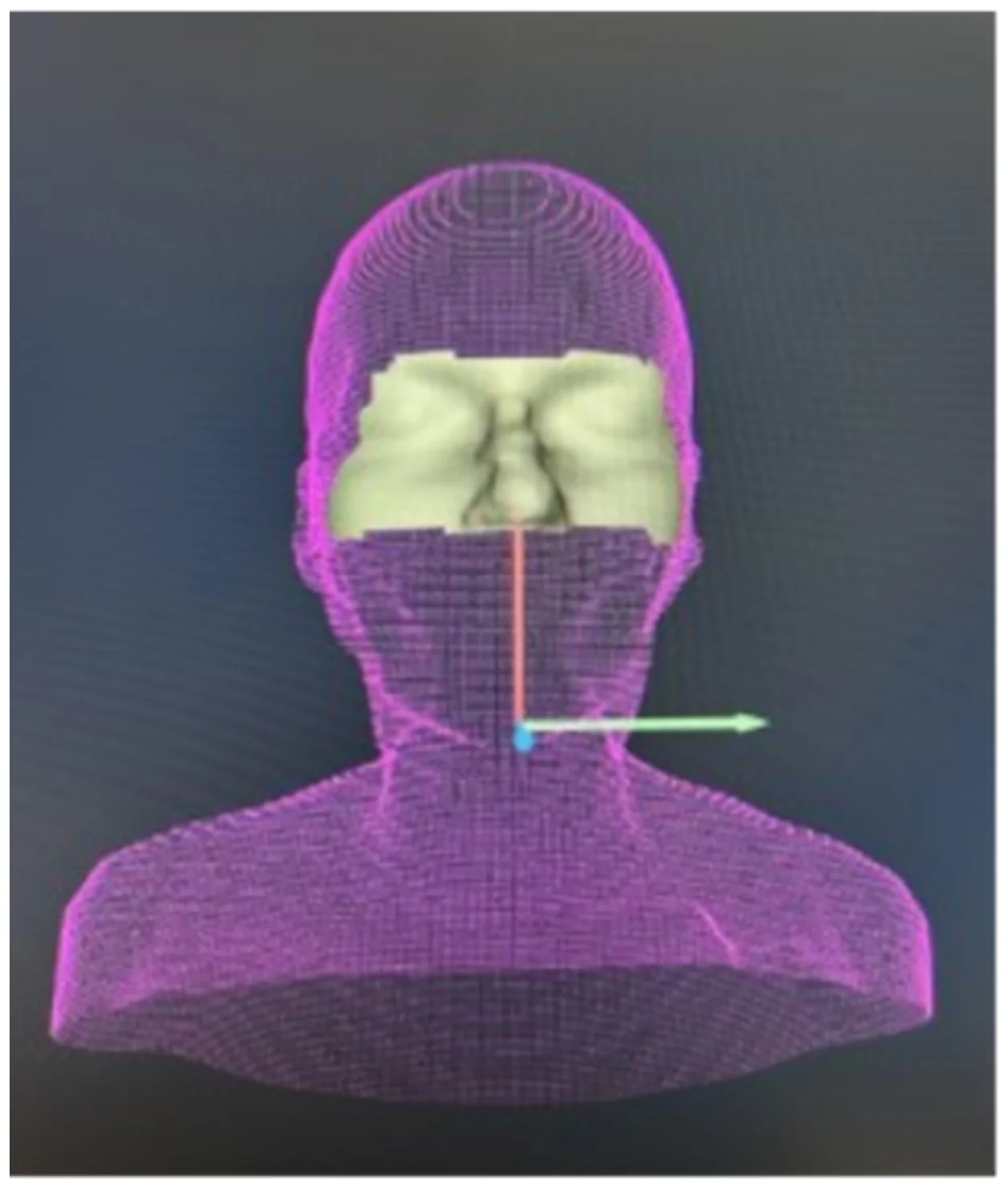
Facial Setup ROI used for initial head positioning. The ROI is defined superiorly by the supraorbital ridge, inferiorly above the mouth, and laterally as far as permitted by the mask aperture.
2. **Monitoring (Isocentric) ROI** (Fig. 4): A larger combined ROI encompassing the visible facial area, lower neck and upper thorax, including the suprasternal notch and clavicles, was created for lower neck and shoulder positioning and subsequent intrafraction monitoring.

**Fig. 4:**
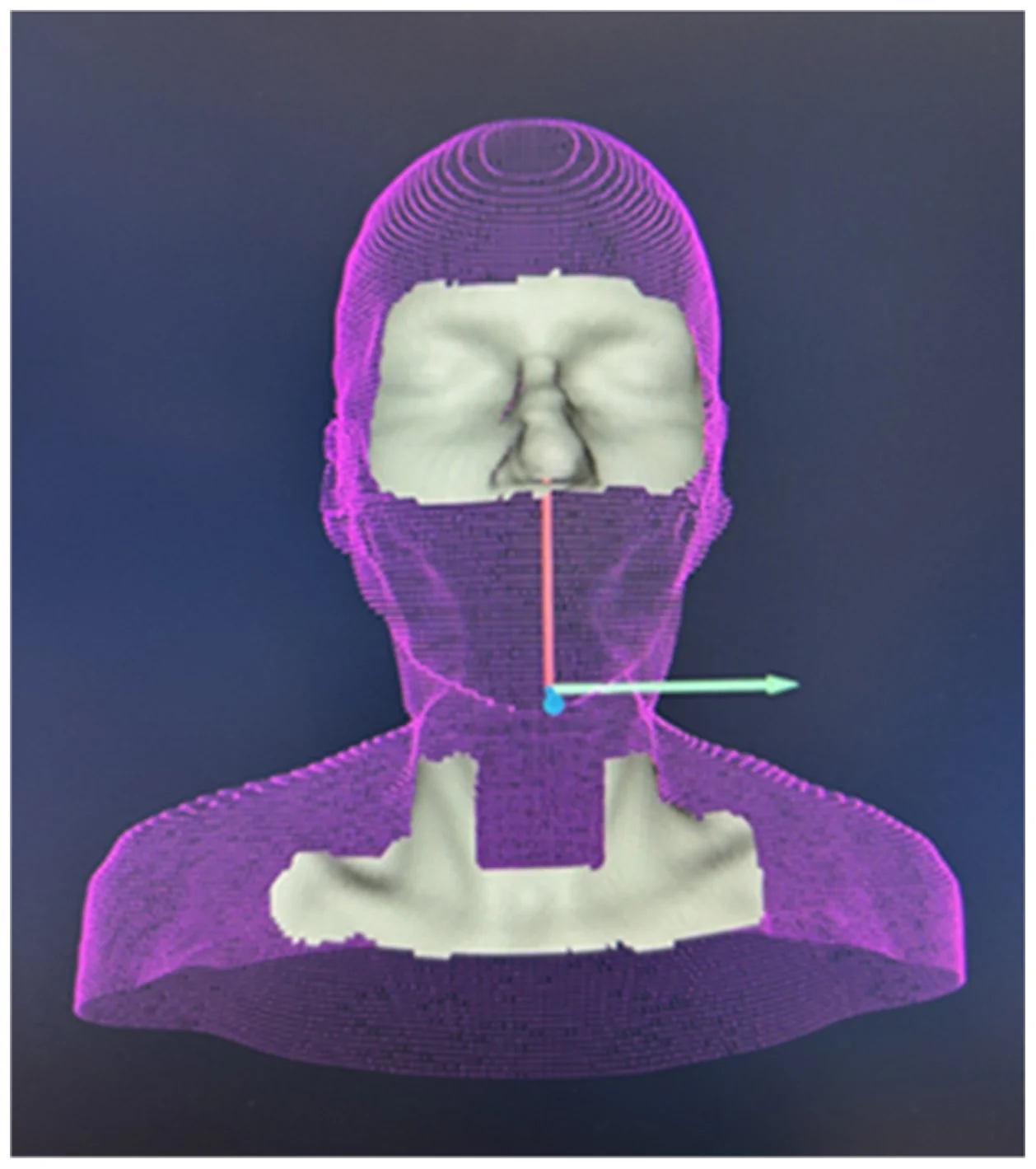
Monitoring (Isocentric) ROI encompassing the face, lower neck and upper thorax, including the suprasternal notch and clavicles. This ROI is used to refine lower neck and shoulder position and for subsequent intrafraction monitoring.

ROIs were initially generated within the AlignRT preparation workspace using the patient surface and anatomical landmarks. These ROIs, particularly the Monitoring ROI, were considered a starting point and personalised according to individual patient and surface characteristics.

ROIs were reviewed during the first treatment fraction and adjusted where required before being adopted for ongoing setup and monitoring. For example, ROI boundaries could be modified to exclude dense facial hair, scar tissue or tattoos where surface tracking was unreliable, and to accommodate tracheostomies or mouth bites. ROI extent was also adapted according to the individual mask opening and the available facial, neck and thoracic surfaces. Where applicable, the appropriate skin-tone setting was selected to optimise surface tracking.

**SGRT setup tolerances and beam-hold thresholds** were set at 2 mm for translational displacement and 2° for rotational displacement. These thresholds were applied during SGRT-guided setup and intrafraction monitoring and were intentionally selected to provide sensitive detection of positional changes before image verification and throughout treatment delivery, while remaining practical for routine clinical use.

***3****.* ***Daily treatment setup*** (Fig. 5)

**Fig. 5:**
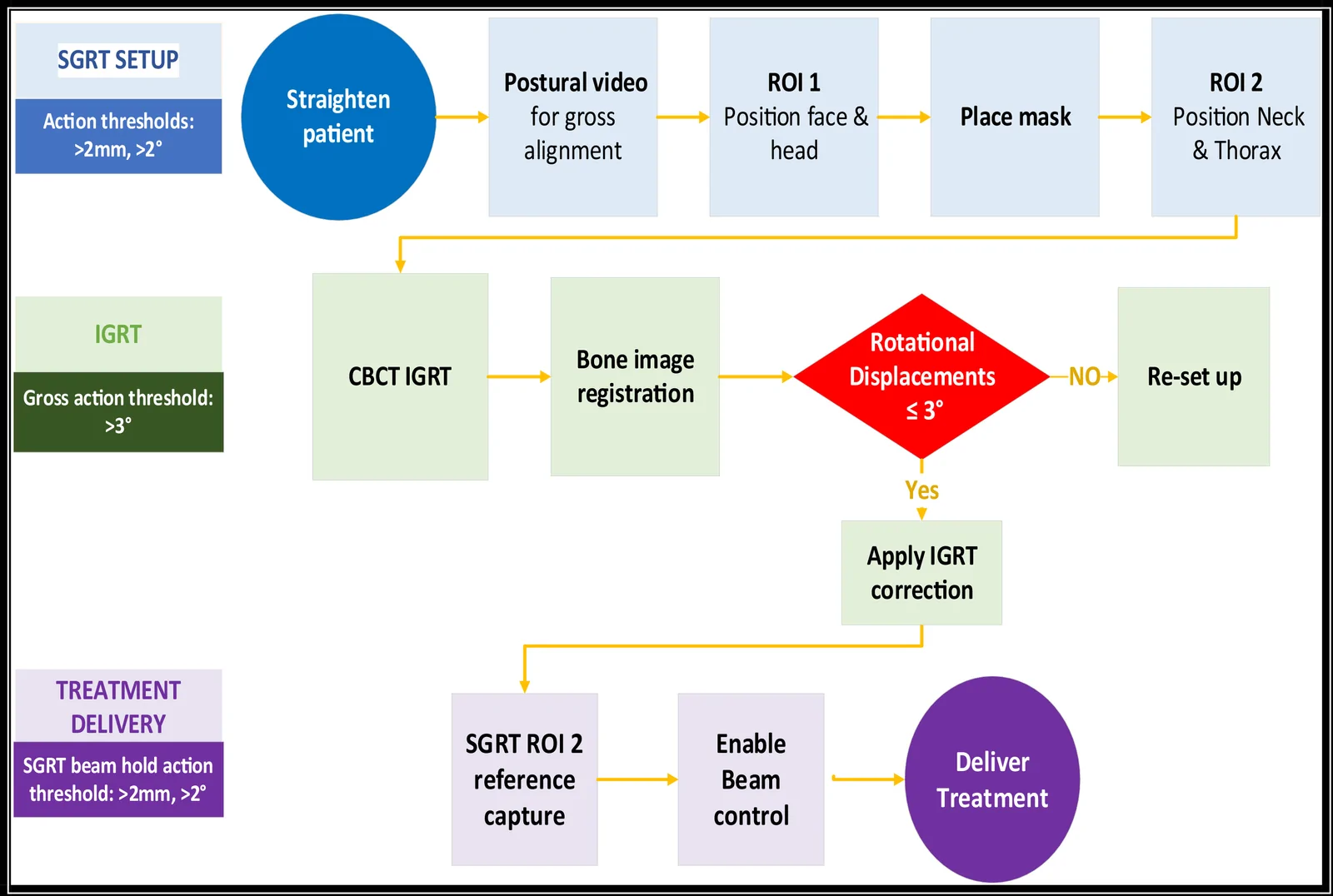
Treatment positioning, image-verification and delivery workflow for patients treated using open-face immobilisation and SGRT, including SGRT setup and beam-hold thresholds (>2 mm/2°) and the IGRT rotational re-setup criterion (>3°).

1. **Initial alignment:** Align the patient using room lasers and the indexed immobilisation equipment. Ensure the knee support is correctly positioned and, for patients using the 3-point open-face mask, position the shoulder retractors according to the simulation setup.
2. **Gross SGRT positioning:** Use SGRT postural video for gross positioning based on the patient’s external contour.
3. **Facial setup using ROI 1:** Use the facial Setup ROI to refine translational alignment, head tilt and rotation.
4. **Secure mask:** Once satisfactory facial alignment is achieved, secure the patient in the thermoplastic mask.
5. **Lower neck and shoulder refinement using ROI 2:** Switch to the Monitoring (Isocentric) ROI encompassing the face, lower neck and upper thorax. Refine the lower neck and shoulder position while maintaining the facial alignment established with ROI 1. Use the deformation display to identify areas requiring correction.
6. **Proceed to IGRT verification:** Once the patient is positioned within the predefined SGRT setup tolerances, proceed to IGRT verification.

***4. IGRT***

All patients underwent daily CBCT for treatment position verification. Registration was performed using bony anatomy according to institutional H&N image-guidance practice.

Following CBCT review, couch corrections were applied. For patients treated on a 6DoF couch, both translational and rotational corrections were applied. For patients treated on couches without 6DoF capability, translational corrections only were applied.

A 3° rotational tolerance was used as the IGRT re-setup criterion for all patients, irrespective of couch capability. Patients were re-positioned if this tolerance was exceeded. This tolerance was considered appropriate within the context of the 3 mm PTV margin applied to all patients in the trial.

Daily image guidance remained an essential component of the protocol. SGRT was not used as a replacement for volumetric image verification, but rather to improve initial setup quality and enable continuous motion monitoring throughout treatment delivery.

***5. Intrafraction monitoring***

After IGRT verification, SGRT monitoring continued until completion of treatment delivery using the larger Isocentric monitoring ROI.

A new SGRT surface reference capture was acquired following IGRT corrections, establishing the patient’s verified treatment position as the baseline for intrafraction monitoring. This enabled real-time detection of displacement relative to the confirmed treatment position and provided an additional safety layer for identifying movement during beam-on.

Monitoring sensitivity can decrease as ROI size increases due to averaging across larger anatomical areas. The selected monitoring ROI was intentionally large enough to assess clinically relevant lower neck and thoracic stability, while not reducing the sensitivity of the facial portion of the ROI.

### Considerations and troubleshooting for reproducing the workflow

The following considerations reflect practical lessons identified during development and implementation of the workflow within the OPEN trial and may assist centres seeking to reproduce the approach.

1. **Use the facial Setup ROI as the primary setup region.** The face provides consistent topography and supports reliable translational and rotational positioning. The workflow deliberately progresses from a small facial ROI to a larger overlapping ROI encompassing the face, lower neck and upper thorax. Retaining the face within the second ROI allows the position of the lower neck and shoulders to be refined relative to the established head position, helping to preserve alignment across the extended H&N treatment region.
2. **Avoid replacing the CT-derived reference surface with daily reference captures**, as these may contain residual rotational or positional offsets. If adopted as the new setup surface, this may result in systematic positional drift, particularly in patients experiencing anatomical change.
3. **Daily CBCT IGRT should remain the gold standard for treatment verification.** SGRT should complement, rather than replace, established image-verification methods in H&N radiotherapy.
4. **Allow time on the first treatment day for ROI personalisation.** Where satisfactory positioning is not achieved or where the deformation is inconsistent with the planned surface, assess the ROI and tailor this as required. Patient-specific factors, including dense facial hair, skin tone, mouth bites, scars, tattoos, tracheostomies, shoulder position and variation in mask opening, may affect tracking quality. ROI boundaries should be adjusted as required to optimise surface capture before routine treatment delivery.
5. **Allow for staff training and workflow familiarisation.** Implementation requires dedicated education and an initial learning period to achieve consistent application, operator confidence and clinical proficiency.
6. **Do not rely on a single large ROI for all stages of H&N setup.** The anatomical length and mobility of the region may allow local positional discrepancies to be masked through averaging across a large surface.
7. **Optimise and standardise shoulder immobilisation when using a 3-point open-face mask.** In this workflow, shoulder retractors were used to reproduce shoulder position in the absence of thermoplastic shoulder fixation. Consistent indexing, patient hand position and retractor tension should be maintained throughout treatment. Additional shoulder immobilisation, such as a mouldable shoulder cushion or indexed hand poles, may also be considered to improve reproducibility.
8. **Anticipate camera occlusion during treatment delivery.** Temporary loss of surface visibility may occur when the treatment gantry or kV imaging equipment obstructs one or more SGRT cameras. ROI design should therefore maximise anatomically distinct surface regions with sufficient topographic variation and visible surface area to support tracking during partial occlusion. In this workflow, individualised ROI design and a **1-second beam-hold delay** reduced unnecessary beam interruptions associated with brief tracking loss. Beam-hold behaviour and ROI performance should be validated locally according to treatment-machine and camera configuration.

## Protocol validation

The open-face SGRT workflow was evaluated within the OPEN Phase III randomised control trial. A total of 230 patients were enrolled, of whom 199 were evaluable for the primary endpoint of setup accuracy: 68 in Arm A (5-point closed-face mask), 71 in Arm B (3-point open-face mask with SGRT) and 60 in Arm C (5-point open-face mask with SGRT). Full trial methodology and outcomes are reported in the OPEN trial manuscript [15]: the key findings relevant to validation of the workflow are summarised here.

**Setup accuracy:** Interfraction setup accuracy was assessed using daily CBCT. Mean deviations across six dimensions were compared using one-way ANOVA with Holm-adjusted p-values, followed by pairwise Welch’s *t*-tests where significant. Bayesian estimation was also used to quantify pairwise differences using 95% Highest Density Intervals (HDIs) [15].

No statistically significant differences were observed between the three immobilisation systems in the vertical, lateral or rotational dimensions. Longitudinal displacement was significantly greater in the closed-mask cohort (Arm A: 1.4 mm) than in either open-mask cohort (Arm B: −0.1 mm; Arm C: −0.5 mm; *p* < 0.001). Although a small statistical difference was observed between the two open-mask groups (*p* = 0.04), Bayesian estimation demonstrated a 95% HDI of 0.03–0.81 mm, indicating limited clinical magnitude. Systematic (Σ) and random (σ) errors were comparable across the three arms (≤0.4 mm), with calculated interfraction margins ranging from 3.4 mm (Arm A, Longitudinal) to 4.4 mm (Arm A, Vertical) across arms and dimensions.

**Intrafraction stability:** A planned interim analysis of the first 56 participants evaluated intrafraction motion using CBCT and continuous SGRT monitoring [16]. Mean CBCT-measured deviations were <0.4 mm translationally and <0.2° rotationally. SGRT monitoring in the open-mask cohorts demonstrated overall 95th percentile deviations of 0.4 mm translationally and 0.8° rotationally. These findings demonstrated intrafraction stability comparable to conventional closed-mask immobilisation, while SGRT also enabled detection of transient motion not captured by CBCT alone.

**Clinical efficiency and resource utilisation:** An initial implementation learning curve was observed. Mean treatment time for patients using the open-face mask and SGRT workflow was approximately 15 min during the first week of a patient’s treatment, reducing to 12 min during subsequent weeks. Across the trial implementation period, mean SGRT setup and treatment time decreased from 16.0 min at the beginning of the trial to 13.1 min towards completion. Following workflow familiarisation, median treatment duration across both open-mask groups remained below the standard institutional 15-minute treatment appointment. These findings relate to routine treatment delivery following implementation; dedicated staff training and workflow familiarisation were required during introduction of the technique [15].

**Replanning:** Across the 230 patients enrolled, 18/230 (7.8%) underwent replanning for mask-related reasons: nine in Arm A, six in Arm B and three in Arm C. Six patients assigned to the closed-mask group required replanning with an open-face mask because of mask intolerance. Excluding intolerance, 12/230 patients (5%) underwent replanning for suboptimal immobilisation (Arm A: *n* = 3; Arm B: *n* = 6; Arm C: *n* = 3) [15].

**Patient experience:** At treatment completion, overall patient experience was significantly more favourable in both open-mask groups than in the closed-mask group (Arm A vs. Arm B: *p* < 0.001; Arm A vs. Arm C: *p* < 0.004). Full patient-reported outcome analyses are provided in the accompanying OPEN trial manuscript [15].

**ROI topography analysis:** A dedicated analysis using topographic metrics was undertaken to assess SGRT ROI quality and robustness. Increasing the open-face mask aperture generally increased the facial surface topography available for tracking, providing greater surface gradients and geometric information. However, the central facial region consistently provided the most clinically relevant and robust topographic information. These findings support the use of relatively small, carefully selected ROIs centred on key facial features and application of the workflow across open-face mask designs with varying aperture sizes [17].

Collectively, these findings provide clinical validation for the workflow described in this MethodsX article, demonstrating reproducible setup accuracy and intrafraction stability while remaining deliverable within standard treatment appointment times following an initial implementation and training period. The present protocol therefore provides the practical implementation companion to the clinical outcomes reported in the OPEN trial [15].

## Limitations

The effectiveness of SGRT may be influenced by patient-specific factors. H&N patients can experience substantial anatomical changes during treatment, including weight loss and tumour regression, which may affect surface-tracking quality and ROI reliability. Individualised ROI design and ongoing clinical assessment are therefore required.

Camera occlusion was identified as a potential limitation of the open-face SGRT workflow. Treatment equipment, such as the gantry or IGRT system, may enter the line of sight between the patient surface and SGRT cameras, reducing the visible surface available for tracking and potentially causing temporary tracking loss or unnecessary beam interruptions. In this study, conducted using C-arm linacs, this was mitigated through individualised ROI design and a 1-second beam-hold delay, allowing brief tracking interruptions to resolve without affecting treatment delivery. The workflow has not been evaluated on bore-type linacs, where camera geometry and surface visibility constraints may differ; further validation would therefore be required in these treatment environments.

For the 3-point open-face mask (Arm B), shoulder retractors were used to reproduce shoulder position in the absence of thermoplastic shoulder fixation. Staff feedback identified occasional setup inconsistencies, and further optimisation of shoulder immobilisation is recommended when reproducing this workflow.

The protocol was developed within a single radiotherapy network using a specific SGRT system and immobilisation equipment. Variations in hardware, software algorithms, camera configuration, and local radiotherapy workflows may affect reproducibility across centres. External validation in different clinical environments is therefore recommended.

Finally, the dual-ROI strategy retains some dependence on manual ROI selection and operator judgement, introducing potential inter-user variability, particularly during early implementation. Structured staff training and standardisation are therefore important to ensure consistent application and clinical confidence in the workflow.

## CRediT authorship contribution statement

**Samantha Ryan:** Conceptualization, Methodology, Validation, Investigation, Writing – original draft, Project administration. **Claire Fitzpatrick:** Writing – original draft, Visualization. **Ciaran Malone:** Conceptualization, Validation, Formal analysis. **Margaret Tynan:** Validation, Writing – review & editing. **Rachel Harwood:** Writing – review & editing. **Clodagh Callagy:** Writing – review & editing. **Ruth Woods:** Conceptualization, Writing – review & editing, Supervision. **Sinead Brennan:** Conceptualization, Writing – review & editing, Supervision. **Jill Nicholson:** Conceptualization, Writing – review & editing, Supervision.

## Declaration of competing interest

The authors declare that they have no known competing financial interests or personal relationships that could have appeared to influence the work reported in this paper.

## Data Availability

The data that has been used is confidential.
